# Supplementary material for: Sequencing of the Complete Mitochondrial Genome of Pingus sinensis (Spirurina: Quimperiidae): Gene Arrangements and Phylogenetic Implications
Source: Genes (Basel). 2021 Nov 8;12(11):1772. doi: 10.3390/genes12111772 (PMC8624427; doi:10.3390/genes12111772)
Supplement: Supplementary file 1 [file genes-12-01772-s001.zip › Table S1.pdf]

genes

MDPI

Table S1. Nucleotide codon usage for 12 protein-coding genes of the mitochondrial genome of *P. sinensis*.

nt	aa	nc	%	nt	aa	nc	%	nt	aa	nc	%	nt	aa	nc	%
TTT	Phe	488	14.3	TCT	Ser2	99	2.9	TAT	Tyr	141	4.1	TGA	Trp	26	0.8
TTC	Phe	19	0.6	TCC	Ser2	6	0.2	TAC	Tyr	8	0.2	TGG	Trp	54	1.6
TTA	Leu2	191	5.6	TCA	Ser2	25	0.7	CAT	His	49	1.4	CGT	Arg	28	0.8
TTG	Leu2	293	8.6	TCG	Ser2	4	0.1	CAC	His	2	<0.1	CGC	Arg	2	<0.1
CTT	Leu1	70	2.1	CCT	Pro	58	1.7	CAA	Gln	23	0.7	CGA	Arg	0	0
CTC	Leu1	3	<0.1	CCC	Pro	6	0.2	CAG	Gln	22	0.6	CGG	Arg	3	<0.1
CTA	Leu1	10	0.3	CCA	Pro	8	0.2	AAT	Asn	75	2.2	AGT	Ser1	113	3.3
CTG	Leu1	16	0.5	CCG	Pro	13	0.4	AAC	Asn	5	0.1	AGC	Ser1	2	<0.1
ATT	Ile	196	5.8	ACT	Thr	78	2.3	AAA	Lys	24	0.7	AGA	Ser1	41	1.2
ATC	Ile	5	0.1	ACC	Thr	4	0.1	AAG	Lys	61	1.8	AGG	Ser1	57	1.7
ATA	Met	55	1.6	ACA	Thr	13	0.4	GAT	Asp	72	2.1	GGT	Gly	122	3.6
ATG	Met	79	2.3	ACG	Thr	6	0.2	GAC	Asp	3	<0.1	GGC	Gly	18	0.5
GTT	Val	225	6.6	GCT	Ala	73	2.1	GAA	Glu	26	0.8	GGA	Gly	23	0.7
GTC	Val	18	0.5	GCC	Ala	21	0.6	GAG	Glu	55	1.6	GGG	Gly	88	2.6
GTA	Val	36	1.1	GCA	Ala	10	0.3	TGT	Cys	60	1.8	TAA	*	8	0.2
GTG	Val	47	1.4	GCG	Ala	11	0.3	TGC	Cys	7	0.2	TAG	*	3	<0.1

nt, nucleotide; aa, amino acid; nc, numbers of codons; *, stop codon; total no. of codons is 3407
