# Supplementary material for: Sequencing of the Complete Mitochondrial Genome of Pingus sinensis (Spirurina: Quimperiidae): Gene Arrangements and Phylogenetic Implications
Source: Genes (Basel). 2021 Nov 8;12(11):1772. doi: 10.3390/genes12111772 (PMC8624427; doi:10.3390/genes12111772)
Supplement: Supplementary file 1 [file genes-12-01772-s001.zip › Table S2.pdf]

genes

Table S2. Nucleotide composition and skewness of the protein-coding genes, tRNAs and rRNAs of mitochondrial genomes of *P. sinensis* and *C. robustus*.

Regions	Size(bp)	T(U)	C	A	G	AT(%)	GC(%)	GT(%)	AT skew	GC skew
Pingus sinensis										
PCGs	10221	50.2	8.6	18.7	22.5	68.9	31.1	72.7	-0.457	0.449
1st codon position	3407	42	9.2	23.9	24.9	65.9	34.1	66.9	-0.275	0.461
2nd codon position	3407	51.4	12.8	16.9	18.9	68.3	31.7	70.3	-0.504	0.194
3rd codon position	3407	57.1	3.8	15.2	23.8	72.3	27.6	80.9	-0.579	0.726
atp6	597	53.4	9.2	16.9	20.4	70.3	29.6	73.8	-0.519	0.379
cox1	1566	46.5	11.1	20.2	22.2	66.7	33.3	68.7	-0.393	0.332
cox2	687	42.9	9.9	21.5	25.6	64.4	35.5	68.5	-0.332	0.443
cox3	768	50.5	9.9	18.1	21.5	68.6	31.4	72	-0.472	0.369
cytb	1110	50.5	9	19.1	21.4	69.6	30.4	71.9	-0.451	0.407
nad1	873	54.8	7	16.8	21.4	71.6	28.4	76.2	-0.53	0.508
nad2	832	52.5	7	16.5	24	69	31	76.5	-0.523	0.55
nad3	327	53.5	4.9	16.5	25.1	70	30	78.6	-0.528	0.673
nad4	1215	50.9	9.2	17.8	22.1	68.7	31.3	73	-0.482	0.412
nad4L	234	53.4	5.1	17.9	23.5	71.3	28.6	76.9	-0.497	0.642
nad5	1584	49.3	7	20.3	23.4	69.6	30.4	72.7	-0.417	0.539
nad6	429	52.7	7.9	17.7	21.7	70.4	29.6	74.4	-0.497	0.465
rrnL	945	42.4	7	30.2	20.4	72.6	27.4	62.8	-0.169	0.49
rrnS	682	35	12.3	30.6	22	65.6	34.3	57	-0.067	0.282
rRNAs	1627	39.3	9.2	30.4	21.1	69.7	30.3	60.4	-0.129	0.391
tRNAs	1238	38.9	9.3	30.8	21	69.7	30.3	59.9	-0.117	0.387
Full genome	13874	46.9	8.9	21.8	22.4	68.7	31.3	69.3	-0.365	0.431
Cucullanus robustus										
PCGs	10269	49	9.7	20.7	20.5	69.7	30.2	69.5	-0.405	0.356
1st codon position	3423	42	9.2	26.6	22.2	68.6	31.4	64.2	-0.225	0.413
2nd codon position	3423	52.5	13.2	17.7	16.6	70.2	29.8	69.1	-0.495	0.114

3rd codon position	3423	52.4	6.8	17.9	22.8	70.3	29.6	75.2	-0.491	0.539
atp6	600	51.5	7.8	18.8	21.8	70.3	29.6	73.3	-0.464	0.472
cox1	1578	43.7	13	21.3	22	65	35	65.7	-0.345	0.257
cox2	696	43.5	11.1	22.6	22.8	66.1	33.9	66.3	-0.317	0.347
cox3	766	46.7	10.2	20.8	22.3	67.5	32.5	69	-0.385	0.373
cytb	1099	48.5	9.6	20.7	21.1	69.2	30.7	69.6	-0.401	0.373
nad1	873	48.5	9.5	19.1	22.9	67.6	32.4	71.4	-0.434	0.413
nad2	843	53.4	7.7	20	18.9	73.4	26.6	72.3	-0.454	0.42
nad3	333	53.8	5.7	23.4	17.1	77.2	22.8	70.9	-0.393	0.5
nad4	1230	48.8	10.8	20.4	20	69.2	30.8	68.8	-0.41	0.298
nad4L	234	51.7	4.3	26.9	17.1	78.6	21.4	68.8	-0.315	0.6
nad5	1584	52.5	8.8	20.3	18.4	72.8	27.2	70.9	-0.441	0.355
nad6	435	54	9	20	17	74	26	71	-0.46	0.31
rrnL	958	46.2	6.1	32.7	15	78.9	21.1	61.2	-0.172	0.426
rrnS	677	38.1	8.6	35.7	17.6	73.8	26.2	55.7	-0.032	0.345
rRNAs	1635	42.9	7.1	33.9	16.1	76.8	23.2	59	-0.116	0.388
tRNAs	1218	38.9	8	34.3	18.7	73.2	26.7	57.6	-0.063	0.399
Full genome	13972	47	9.1	24.6	19.2	71.6	28.3	66.2	-0.312	0.358
