# Supplementary figures and images for: Sequencing of the Complete Mitochondrial Genome of Pingus sinensis (Spirurina: Quimperiidae): Gene Arrangements and Phylogenetic Implications
Source: Genes (Basel). 2021 Nov 8;12(11):1772. doi: 10.3390/genes12111772 (PMC8624427; doi:10.3390/genes12111772)

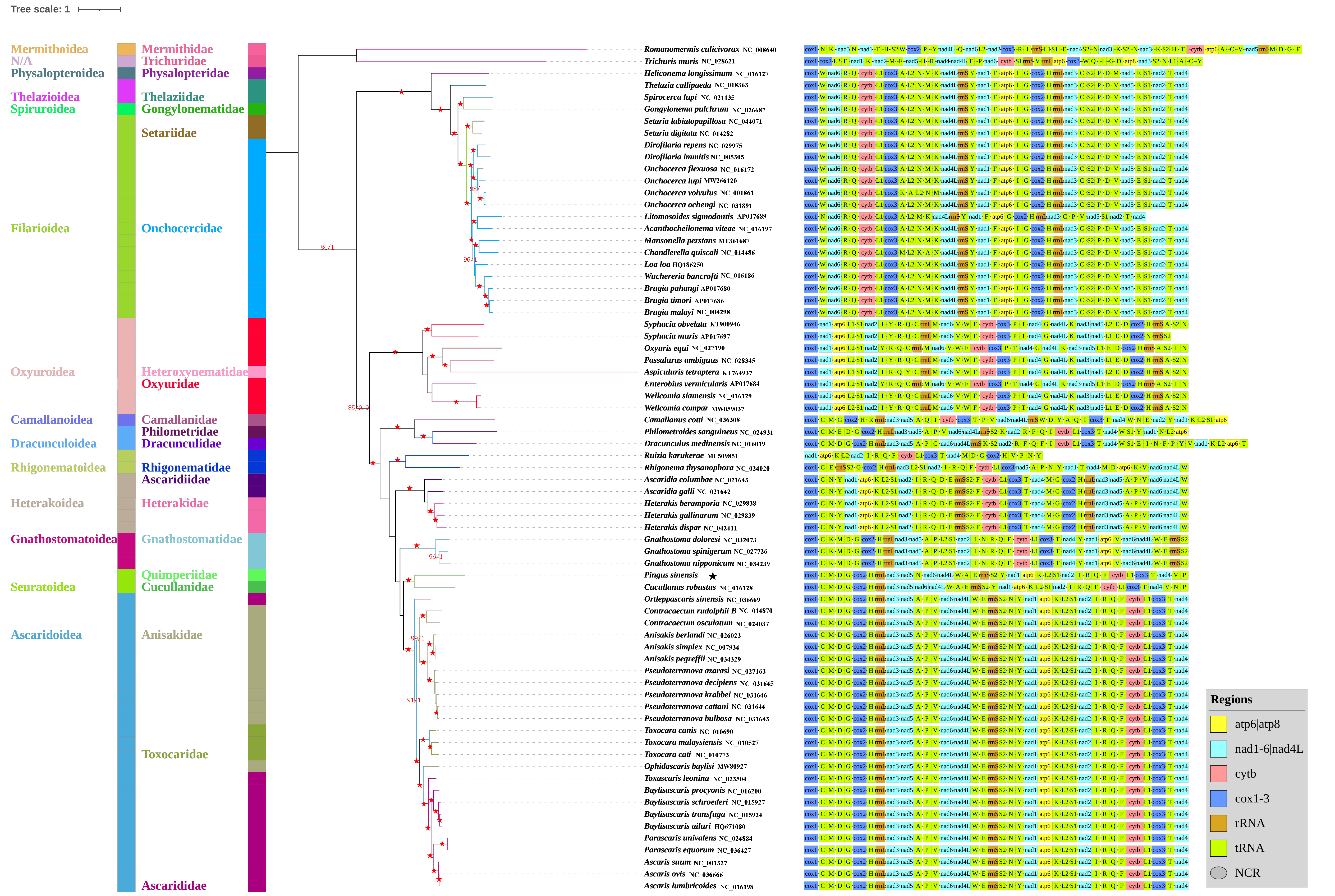

Supplement: Supplementary file 1 [file genes-12-01772-s001.zip › Figure S1.tif]

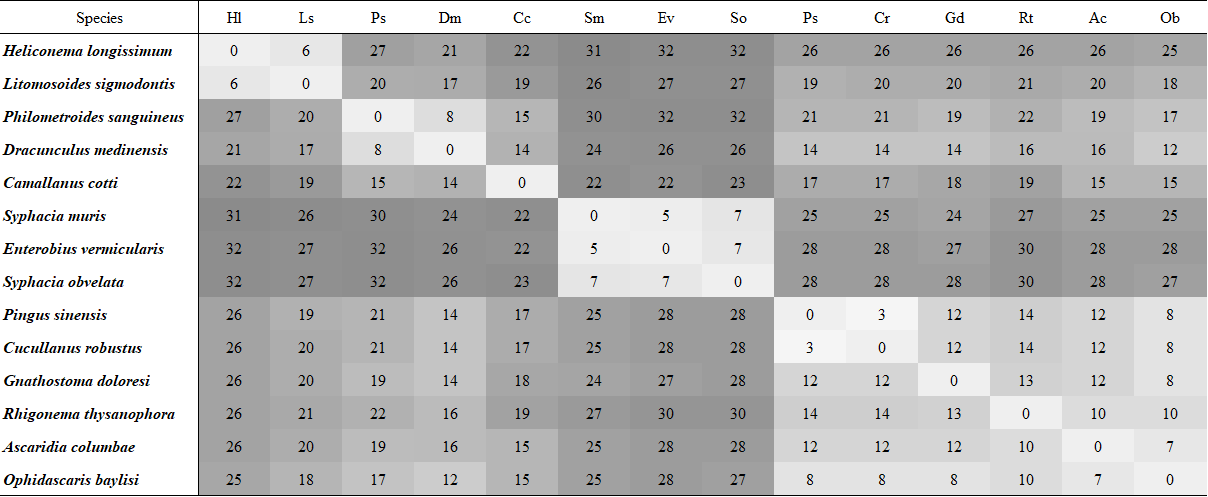

Supplement: Supplementary file 1 [file genes-12-01772-s001.zip › Figure S2.tif]

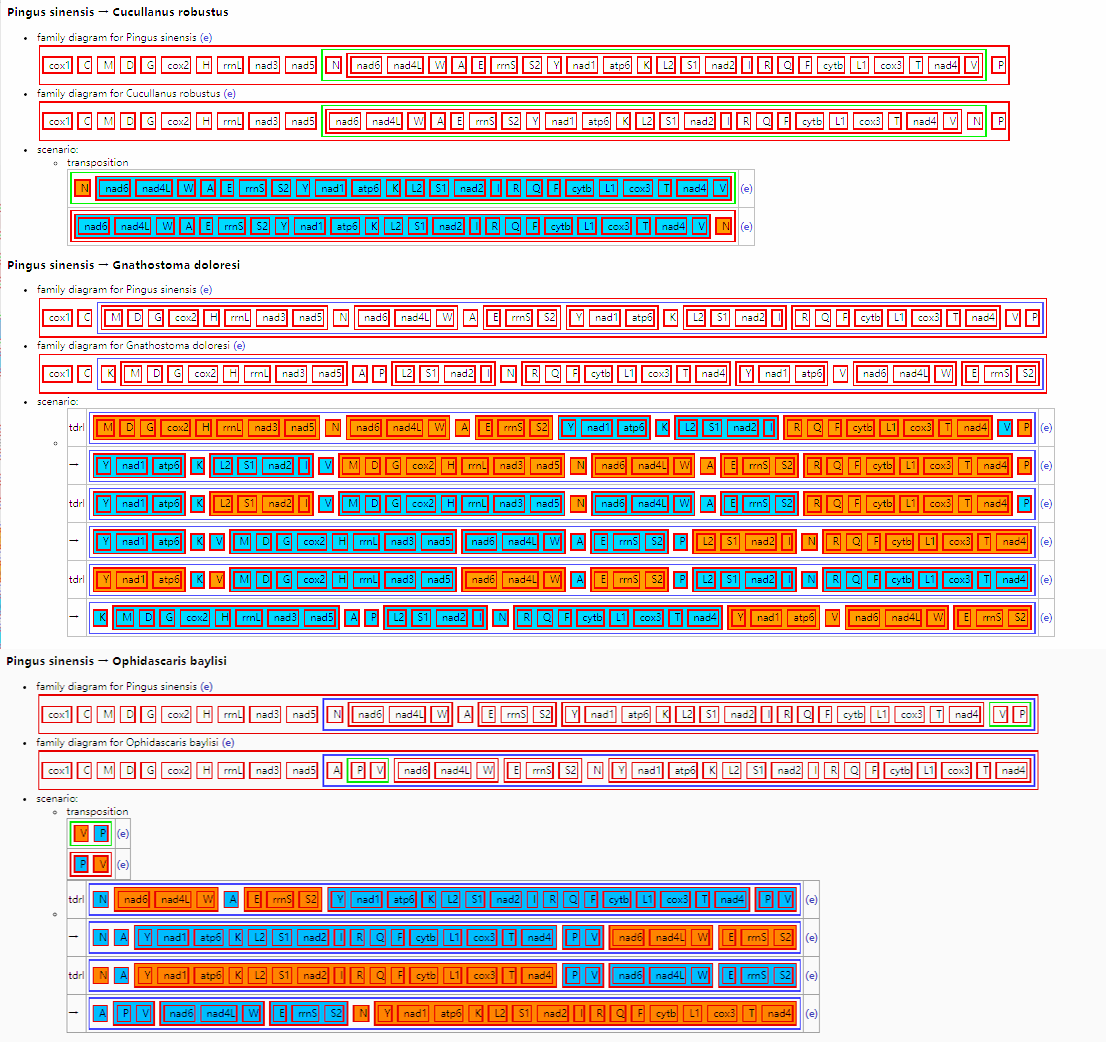

Supplement: Supplementary file 1 [file genes-12-01772-s001.zip › Figure S3.tif]

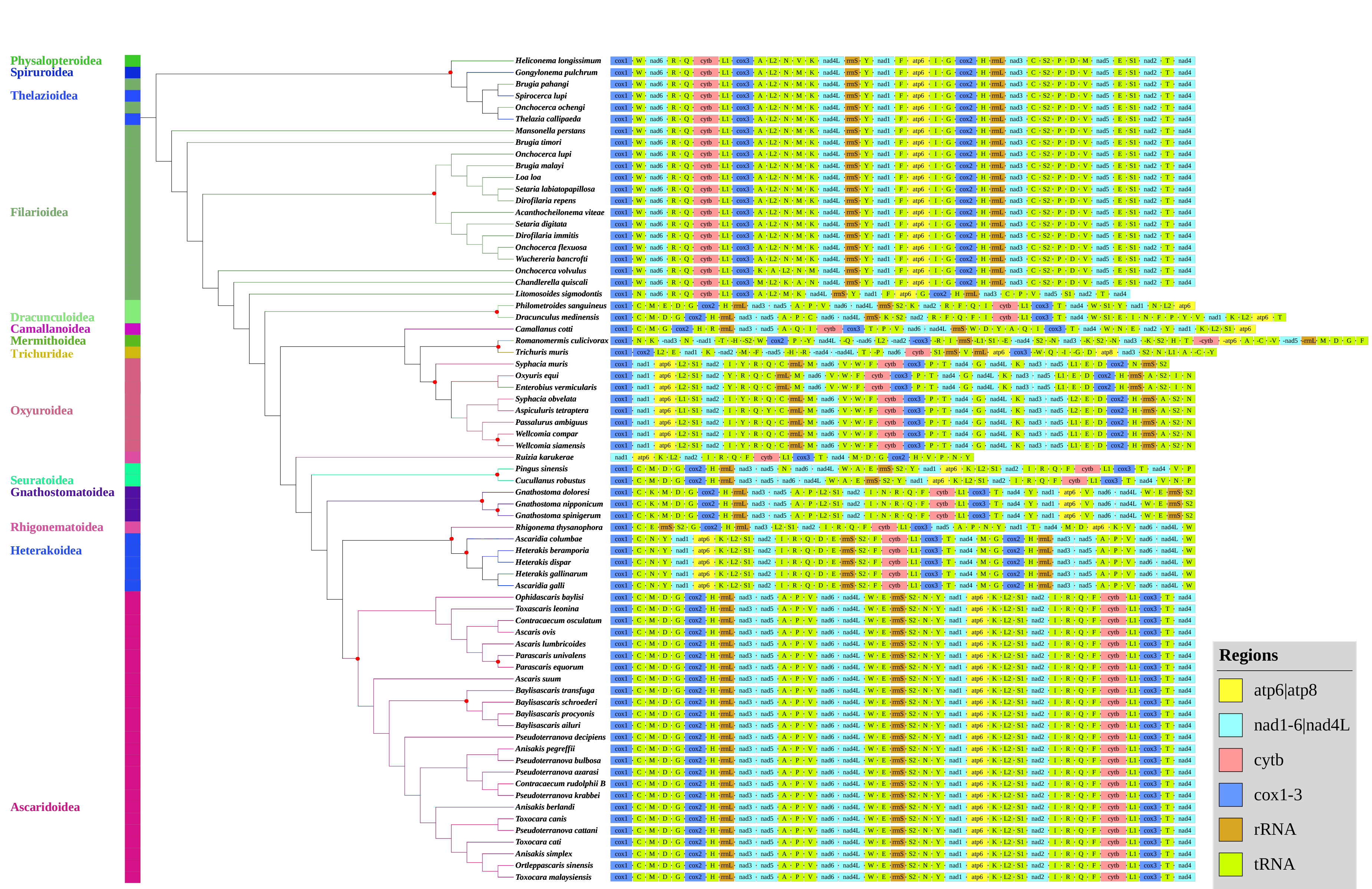

Supplement: Supplementary file 1 [file genes-12-01772-s001.zip › Figure S4.tif]
